# Ethylene facilitates boil‐peeling in fruits

**DOI:** 10.1002/fsn3.1098

**Published:** 2019-08-17

**Authors:** Satoru Murakami, Kazuki Yamaguchi, Nozomi Hashimoto

**Affiliations:** ^1^ Shizuoka Research Institute of Agriculture and Forestry Fruit Tree Research Center Shizuoka Japan

**Keywords:** climacteric fruits, ethylene, heat treatment, peeling, polygalacturonase

## Abstract

Boil‐peeling is a common method of cooking or processing some horticultural crops. While boil‐peeling is possible in some horticultural crops, a comprehensive list of crops for which boil‐peeling is possible does not exist. According to a previous study, ethylene facilitates boil‐peeling of kiwifruits. Thus, we studied the effect of ethylene treatment on boil‐peeling in the kiwifruit variety “Rainbow red.” We found that with increasing ethylene concentration in the fruits, boil‐peeling success of kiwifruits increased. In the no‐ethylene treatment, flesh firmness of the fruits decreased and boil‐peeling could not be carried out successfully. Thus, it was clear that ethylene facilitates boil‐peeling in kiwifruit. Furthermore, boil‐peeling was possible after ethylene treatment in persimmon and Japanese pear, which had proved to be impossible so far. Kiwifruits, persimmon, and Japanese pear were classified as climacteric fruits that react with high ethylene sensitivity. Thus, ethylene may facilitate boil‐peeling in climacteric fruits. This finding can possibly suggest new application for ethylene during fruit processing or in processed fruits.

## 1 INTRODUCTION

Fruit peeling increases the cost of processed fruit products because of the complicated procedure necessary at the processing plants. For this reason, various fruit peeling methods involving agents such as heat treatment, chemical treatment, enzymes, and mechanical devices have been considered. Chemical peeling fruit tastes appear due to the aggressive chemical treatment that the fruit undergoes (Ben‐shalon, Levi, & Pinto, 1986; Coll, 1996). In enzymatic peeling, as the enzyme activity in the fruits remains, the flesh easily crumbling especially canned and preserved citrus (*Citrus unshiu* [Swingle] Marcow.). Boil‐peeling involves steeping the fruits for 30 s in near boiling water (95°C), and quick cooling in iced water, followed by skinning or peeling by hand. Boil‐peeling is a common method of cooking or processing tomatoes (*Lycopersicon esculentum* Mill.), pears (*Pyrus communis* L.), and kiwifruits (*Actinidia chinennsis* Planch.). This traditional method does not use chemicals or enzymes, and the peeled fruit does not affect the chemicals or remaining enzyme. However, this process is difficult to adopt for processing other fruits such as persimmon (*Diospyros kaki* Thunb.), Japanese pear (*Pyrus pyrifolia* (Burm.f.) Nakai). However, a comprehensive list of fruits for which boil‐peeling is possible does not exist. In a recent study, we showed that it is easier to skin kiwifruits by boil‐peeling‐induced premature ripening under the influence of ethylene (Murakami, Yamaguchi, Sasaki, & Noguchi, 2016). However, some mature kiwifruits are hard to skin by boil‐peeling. Interestingly, these fruits do not have the distinctive fragrant flavor characteristic of ethylene‐treated fruits. In climacteric fruit such as kiwifruit, the exponential increase in ethylene production coincides with a rise in respiration by ethylene and correlates with the development of the unique fruit flavor composition (Knee, 1993; Yang & Hoffman, 1984). Also, Murakami, Ikoma, and Yano (2015) suggested that the process of kiwifruit core firmness reduction differs in an ethylene induction‐dependent and an ethylene‐independent manner. From these results, we hypothesized that ethylene facilitates boil‐peeling in kiwifruits, and we investigated the influence of ethylene treatment on the boil‐peeling of kiwifruits. Here, we show that ethylene promotes boil‐peeling in kiwifruits. Furthermore, the same phenomenon was confirmed in persimmon and Japanese pear as well. We report these results and the evaluations in this article.

## 2 MATERIALS AND METHODS

### 2.1 Effect of ethylene treatment on boil‐peeling in the kiwifruit variety “Rainbow red”

**Figure 1:**
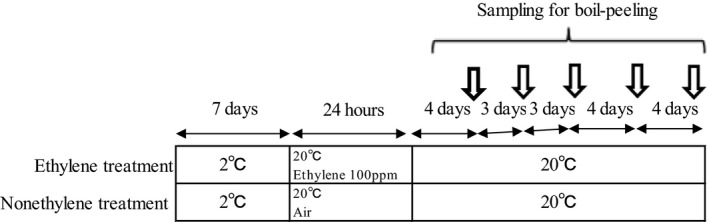
Schematic representation of experimental design to test the effect of ethylene treatment on boil‐peeling of kiwifruit variety ‘Rainbow Red’. After harvest, fruits were either treated or not treated with ethylene at 2 °C for 7 days. A portion of the fruits was used for ethylene (100 ppm) treatment at 20 °C for 24 h; fruits were stored at 20 °C in ambient air. Fruits were investigated at the points indicated by arrows

**Figure 2:**
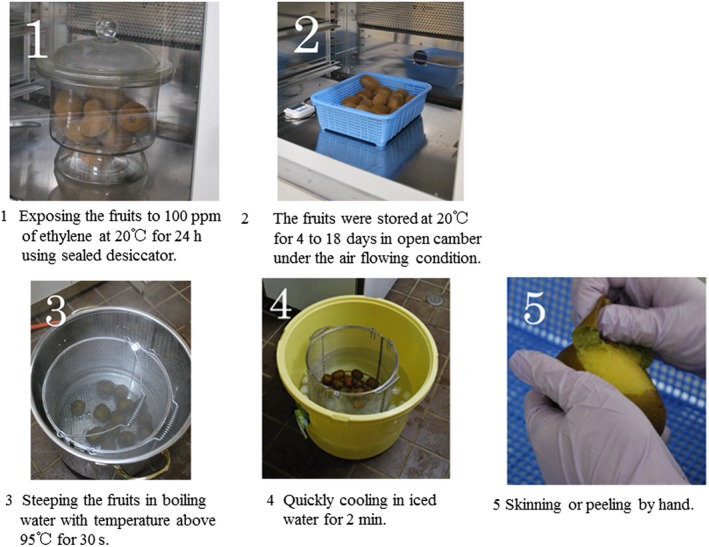
The order of ethylene treatment and Boil‐peeling in this study

**Figure 3:**
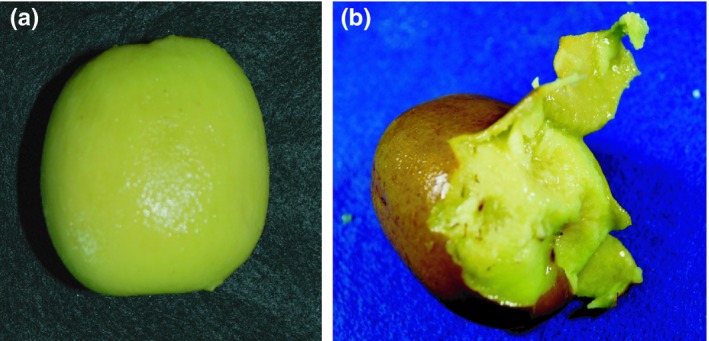
Boil‐peeling success (A) and failure (B) in kiwifruits

Kiwifruit ripening is inducible by either ethylene treatment or low temperature (Mworia et al., 2012). Low temperature modulates fruit ripening in an ethylene‐independent manner (Mworia et al., 2012). This modulation is particularly evident in red or yellow kiwifruit including the variety “Rainbow red” (Asiche et al., 2016). Also, we have previously confirmed that “Rainbow red” kiwifruit ripens on vines with no relation to ethylene (Murakami et al., 2015). Therefore, we used “Rainbow red” kiwifruit in this study. The experimental design consisted of two treatments: one with no ethylene and one with ethylene. The “Rainbow red” fruits were harvested on 30 September 2015 from the experimental orchard at Shizuoka, Japan. Kiwifruits selected for the experiment were similar in size and maturity. In “Rainbow red” kiwifruits, low temperature storage (4°C for 8 days) enhances ethylene sensitivity (Murakami, Ikoma, & Yano, 2014). For this reason, the kiwifruits were immediately transported and stored in a refrigerator at 2°C for 7 days. The fruits were then ripened with and without ethylene treatment (Figure 1). The order of ethylene treatment and boil‐peeling in this study are presented in Figure 2. Hyodo and Fukasawa (1985) and Hyodo, Aizawa, and Ao (1987) suggested that threshold concentration for initiation of autocatalytic ethylene at 21°C is 1 ppm. In this study, the treatment with ethylene condition was above the report conducted by exposing the fruits to 100 ppm of ethylene at 20°C for 24 hr, while in the no‐ethylene treatment, the fruits were stored in an ethylene‐free atmosphere at 20°C for 24 hr. After each treatment, in order to study from immature to overripe fruits, the fruits were allowed to ripen at 20°C for 4 to 18 days. After determining the ripeness of the fruits by measuring the amount of ethylene in the fruits and the nondestructive flesh firmness of the fruits, the kiwifruits were boil‐peeled. Boil‐peeling was performed following this process: steeping the fruits in boiling water with temperature above 95°C for 30 s and next, quickly cooling in iced water, followed by skinning or peeling by hand. To determine whether boil‐peeling was successful or not, specific fruit samples were peeled by hand. Successful fruit peeling involved neat pulling‐off of the skin by application of weak pressure without flesh collapsing (Figure 3). Contrarily, unsuccessful peeling was characterized by exertion of greater pressure, which caused flesh collapse.

### 2.2 Ethylene measurement and nondestructive flesh firmness measurement

Ethylene measurements were conducted by incubating individual fruits in a 250‐ml plastic container as previously reported (Murakami et al., 2014). After 1 hr, 1 ml of headspace gas was withdrawn and injected onto a gas chromatograph (GC‐2014; Shimadzu) equipped with a flame ionization detector (set at 120°C) and an activated alumina column (set at 120°C). We measured flesh firmness by detecting the viscoelastic properties using laser Doppler method, a nondestructive analytical technique (Muramatsu et al. 1999). This method is sufficiently accurate to determine flesh firmness of the “Rainbow red” kiwifruits (Murakami, 2017). The elastic property of the fruit flesh, which represented its firmness, was calculated as *m* ^2/3^ *f* ^2^ (Cooke, 1970, 1972; Yamamoto & Haginuma, 1984), where *f* is a second resonant frequency, and *m* is the fruit mass.

### 2.3 Effect of ethylene treatment on boil‐peeling of persimmon and Japanese pear

It was clear that ethylene facilitates boil‐peeling of kiwifruits. In persimmon and Japanese pear, boil‐peeling is not generally possible, and the effective peeling method is not yet developed. Next, an experiment was performed on the effects of ethylene treatment on persimmon and Japanese pear. We used “Fuyu” persimmon harvested on 5 November 2016 from the Hamamatsu orchard, and Japanese pear “Hosui” harvested on 24 August from the Shizuoka orchard. “Hosui” was reported to be classified as nonclimacteric cultivar (Tian, Hewett, & Lill, 1992). The “Fuyu” persimmon cultivar is hard to peel by enzymatic treatment. Persimmons and Japanese pears selected for the experiment were uniform in size and maturity. Naturally, the ethylene treatment condition for boil‐peeling was unknown in persimmon and Japanese pear, and the condition was performed slightly long in the case of “Rainbow red.” Ethylene treatment was performed followed immediately after harvest, and persimmon was exposed to 100 ppm ethylene at 20°C for 0–2 days; in the no‐ethylene treatment, the fruits were exposed to ethylene‐free air at 20°C for 0–2 days. Japanese pear fruits were exposed to 100 ppm ethylene at 20°C for 0–4 days, while in the no‐ethylene treatment, the fruits were exposed to ethylene‐free air at 20°C for 0–4 days. After ethylene or ethylene‐free treatment, the persimmons and Japanese pears were boil‐peeled, and similar criteria as those adopted to determine the success of boil‐peeling in the case of the kiwifruits were adopted for persimmons and Japanese pears.

## 3 RESULT AND DISCUSSION

**Figure 4:**
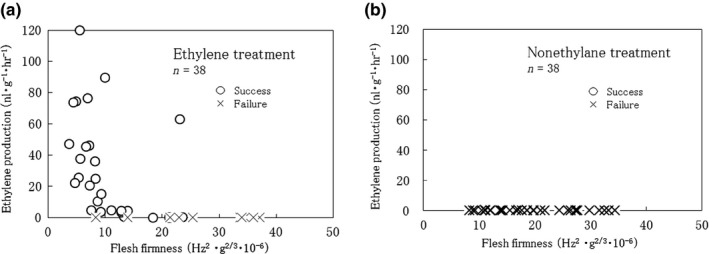
Relationship between Boil‐peeling success or failure and fruit characters (ethylene production and flesh firmness) in kiwifruit

In the ethylene‐treated kiwifruits, the elastic property of the fruit flesh tissue decreased gradually with increase in ethylene production (Figure 4). Terasaki, Sakurai, Yamamoto, Wada, and Nevins (2001) reported after ethylene treatment fruit flesh elasticity decreased exponentially in kiwifruit. This study result was similar to the Terasaki et al. (2001) reported result. Contrarily, the elastic property of the fruit flesh in the no‐ethylene treatment fruits decreased, but there was no ethylene production in the fruits (Figure 4). This phenomenon close to Mworia et al. (2012) reported ripening in an ethylene‐independent manner or “Rainbow red” kiwifruit ripening on vines (Murakami et al., 2015); thus, it seems clear afresh that “Rainbow red” kiwifruits are ripening without ethylene treatment.

**Figure 5:**
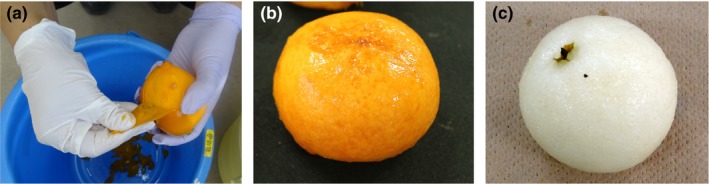
Boil‐peeling in persimmon and Japanese pear. A The state of Boil‐peeling 2 days after ethylene treatment in persimmon (n=14). B External appearance of boil‐peeled persimmon fruit 2 days after ethylene treatment (n=14). C External appearance of boil‐peeled Japanese pear fruit 4 days after ethylene treatment (n=5)

In the ethylene treatment, 28–38 fruits were able to boil‐peeling (Figure 4). Boil‐peeling in the ethylene‐treated fruits was successful; the elastic property of the fruit flesh was around 15 m^2/3^ f^2^, and ethylene production remarkably increased. In the no‐ethylene treatment, trial 38, boil‐peeling was not all successful for all fruits, even if the elastic property of the fruit flesh decreased. In persimmon, boil‐peeling was successful only after 2 days of ethylene treatment (Figure 5); however, it was unsuccessful in the no‐ethylene treatment (Table 1). In Japanese pear, boil‐peeling was successful after 4 days of ethylene treatment (Figure 5), but was unsuccessful in the no‐ethylene treatment or even after two days of ethylene treatment. Thus, it was clear that ethylene can facilitate boil‐peeling in kiwifruit, persimmon, and Japanese pear. In some climacteric fruits, ethylene plays an important role in the initiation and continuation of ripening. A typical climacteric fruit responds to ethylene treatment, which induces rapid changes in color, aroma, and sweetness. However, further examples of uses of ethylene for fruit peeling and processing have not been reported. The results of this study can possibly introduce new applications of ethylene in fruit processing.

**Table 1:** Difference in ethylene or nonethylene treatment period influences successful boil‐peeling in kaki and Japanese pear

Treatment period^a^ (day)	Persimmon		Japanese pear	
	Ethylene treatment	Nonethylene treatment	Ethylene treatment	Nonethylene treatment
0	0/10^b^	0/10	0/5	0/5
2	14/16	0/16	0/5	0/5
4	—c	—	5/5	0/5

a Ethylene treatment condition is exposed in 100 ppm ethylene at 20°C, and nonethylene treatment condition is exposed in air at 20°C.

b The number of boil‐peeling successful fruits/the number of test fruits.

c Without conducted.

Maotani, Kawase, Kamuro, and Hirai (1983) reported that ethylene treatment enhanced peel puffing in Satsuma mandarin. Du et al. (2016) showed that ethylene influences fruit peel tissue at the proteomic level in bananas. Thus, exogenous ethylene treatment should have effects on certain changes in the fruit peel tissue. In some crops, ethylene stimulated the synthesis of some enzymes that cause changes in the cell wall structure, including polygalacturonase and pectin methylesterase (Chen, Liu, Li, Li, & Yuan, 2017; Grierson & Tucker, 1983). Polygalacturonase is one of the hydrolases that destroy cell wall structure (Fischer & Bennett, 1991). Consequently, polygalacturonase activation in fruit peel tissue, in particular, may be listed as a factor that induces changes that facilitate boil‐peeling. It is, however, necessary to study the effects of ethylene on fruit peel tissue by various analyses involving chemistry, gene expression, and immunohistochemistry.

There was no information to the detailed fruit quality difference between ethylene‐treated fruit and nontreated fruits in this study. Mworia et al. (2012) reported increases in the sugar content and reduction in citric acid content were accelerated in response to ethylene, while nontreated fruits showed slow but steady changes similar to those observed in ethylene‐treated fruits. However, Murakami, Yamaguchi, Sasaki, and Noguchi (2019) reported the detailed boil‐peeled fruit quality; flesh firmness and contents of sugar, organic acid, and ascorbic acid were not different between boil‐peeling and knife‐peeling methods. Thus, it was considered that ethylene treatment and boil‐peeling have little effects on the fruit quality. However, there has been a relatively little study on the boil‐peeling, and more detailed investigations were necessary to gain the understanding of the processing method.

In this study, we confirmed that boil‐peeling is possible after ethylene treatment in kiwifruit, persimmon, and Japanese pear. Boil‐peeling is a common method of cooking or processing tomato and pear. These fruits can be classified as climacteric fruit that reacts with high sensitivity to ethylene. Both climacteric and nonclimacteric Japanese pear cultivars exist (Kitamura, Iwata, Fukushima, Furukawa, & Ishiguro, 1981; Tanabe, Tamura, & Itai, 1994). “Hosui” we used in this study was reported as nonclimacteric cultivar (Tian et al., 1992). Nonclimacteric fruits autocatalytically rise in ethylene evolution but reveal ripening‐related responses to exogenous ethylene (Goldschmidt, 1997). Thus, ethylene may facilitate boil‐peeling regardless of climacteric fruits. Therefore, further investigations should identify whether ethylene promotes boil‐peeling in climacteric or nonclimacteric fruits. Also, in this study, we confirmed the differences in the duration of ethylene treatment required by persimmon and Japanese pear (Table 1). It is anticipated that ethylene treatment conditions to facilitate boil‐peeling are different among crops. For this reason, detailed ethylene treatment conditions including ethylene concentration, duration of treatment, and temperature during treatment in practical terms should be investigated in future.

## 4 CONCLUSION

We hypothesized that ethylene facilitates boil‐peeling in kiwifruits, and we investigated the influence of ethylene treatment on the boil‐peeling of kiwifruits. We confirmed that boil‐peeling is possible after ethylene treatment in kiwifruit. The same phenomenon was confirmed in persimmon and Japanese pear as well. These results of this study can possibly introduce new applications of ethylene in fruit processing.

## CONFLICT OF INTEREST

The authors declare that there was no conflict of interests.

## ETHICAL STATEMENT

This study does not involve any human or animal testing.
